# Risks of cancers for carriers of monoallelic *MUTYH* mutation with a family history of colorectal cancer

**DOI:** 10.1186/1897-4287-9-S1-P40

**Published:** 2011-03-10

**Authors:** Aung K Win, Sean P Cleary, James G Dowty, Noralane M Lindor, Polly A Newcomb, Robert W Haile, Joanne P Young, Daniel D Buchanan, Loïc Le Marchand, Roger Green, John L Hopper, Steven Gallinger, Mark A Jenkins

**Affiliations:** 1 Centre for Molecular, Environmental, Genetic and Analytic Epidemiology, The University of Melbourne, Parkville, Victoria 3010 Australia; 2 Samuel Lunenfeld Research Institute, Mount Sinai Hospital, Toronto, Ontario, Canada; 3 Cancer Care Ontario, Toronto, Ontario, Canada; 4 Department of Medical Genetics, Mayo Clinic, Rochester, Minnesota, USA; 5 Cancer Prevention Program, Fred Hutchinson Cancer Research Center, Seattle, Washington, USA; 6 Familial Cancer Laboratory, Queensland Institute of Medical Research, Herston, Brisbane Q4006 Australia; 7 Department of Preventive Medicine, University of Southern California, Los Angeles, California, USA; 8 Cancer Research Center, University of Hawaii, Honolulu, Hawaii 96813 USA; 9 Memorial University of Newfoundland, St. John's, Newfoundland, Canada

## Background

Several studies have shown an increased risk of colorectal and extracolonic cancers for carriers of germline *MUTYH* mutations inherited from both parents (biallelic mutations). Extracolonic cancer risks for carriers of a *MUTYH* mutation inherited from only one parent (monoallelic mutation) have not previously been estimated.

## Materials and methods

We identified 144 families of *MUTYH* mutation carriers from three countries that we ascertained through population-based sources of the multi-site, international Colon Cancer Family Registry. Mutation status, sex, age, and histories of cancer, polypectomy, and hysterectomy were sought from 2,179 of their relatives. Using Cox regression weighted to adjust for the method of ascertainment, we estimated the country-, age- and sex-specific standardized incidence ratios (SIRs) of colorectal and extracolonic cancers for monoallelic mutation carriers, compared with the general population, and corresponding age-specific cumulative risks.

## Results

Monoallelic mutation carriers with a family history of CRC had a significantly increased incidence of CRC (SIR = 2.04; 95% confidence interval, CI = 1.56 – 2.70; *P* <0.001), gastric cancer (SIR = 3.24; 95% CI = 2.18 – 4.98; *P* <0.001), and endometrial cancer (SIR = 2.23; 95% CI = 1.13 – 4.86; *P* = 0.03) and a marginal increased incidence of liver cancer (SIR = 3.09; 95% CI = 1.07 – 12.25; *P* = 0.07) compared to the general population. The estimated cumulative risks to age 70 years based on the population cancer incidence of the United States were as follows: for CRC, 6% (95% CI = 5 – %) for men and 4% (95% CI = 3 – 6%) for women; for gastric cancer, 2% (95% CI = 1 – 3%) for men and 0.7% (95% CI = 0.5 – 1%) for women; for liver cancer, 1% (95% CI = 0.3 – 3%) for men and 0.3% (95% CI = 0.1 – 1%) for women; and for endometrial cancer, 4% (95% CI = 2 – 8%). There was no evidence of increased risks for cancer of the brain, pancreas, kidney, lung, breast or prostate.

## Conclusion

Monoallelic *MUTYH* mutation carriers with a family history of CRC are at increased risk of colorectal, gastric, liver and endometrial cancers.

